# Alternatively spliced ANLN isoforms synergistically contribute to the progression of head and neck squamous cell carcinoma

**DOI:** 10.1038/s41419-021-04063-2

**Published:** 2021-08-03

**Authors:** Erliang Guo, Xionghui Mao, Xueying Wang, Lunhua Guo, Changming An, Cong Zhang, Kaibin Song, Guohui Wang, Chunbin Duan, Xiwei Zhang, Xianguang Yang, Zhennan Yuan, Ji Sun, Xiaomei Li, Weiwei Yang, Hongxue Meng, Susheng Miao

**Affiliations:** 1 Department of Surgery, The 2nd Affiliated Hospital of Harbin Medical University, Harbin, 150081 China; 2 Department of Head and Neck Surgery, Harbin Medical University Cancer Hospital, Harbin, 150081 China; 3 Department of Head and Neck Surgery, Chinese National Cancer Center & Chinese Academy of Medical Sciences Cancer Hospital, Beijing, China; 4 Department of Pathology, Harbin Medical University Cancer Hospital, Harbin, 150081 China; 5 Department of Pathology, Harbin Medical University, Harbin, 150081 China

**Keywords:** Cancer metabolism, Cancer microenvironment, Head and neck cancer, Metastasis

## Abstract

Head and neck squamous cell carcinoma (HNSCC) is a common cancer with high mortality. Anilin actin-binding protein (ANLN) has been reported to be associated with carcinogenesis in multiple tumors. However, the expression pattern and functional effects of ANLN in HNSCC remain to be unclear. Clinical data and online databases were used to analyze the expression of ANLN and its relationship with HNSCC patient survival. Expression of two major splice variants of ANLN was assessed in HNSCC tissues and cell lines. The functional effects and related mechanisms of ANLN isoforms were investigated in HNSCC in vitro and in vivo. Our study showed that patients with high expression of ANLN had a poor prognosis. The two primary isoforms of ANLN transcripts ANLN-201 and ANLN-210 were highly expressed in HNSCC tissues and cell lines. Knockout of ANLN restrained cell proliferation, migration, and invasion of SCC-9 cells. Mechanically, ANLN-201 could interact with c-Myc to keep its protein stability, thereby playing a oncogenic role in HNSCC. ANLN-210 could be transferred to macrophages via exosomes by binding to RNA-binding protein hnRNPC. Exosomal ANLN-210 promoted macrophage polarization via PTEN/PI3K/Akt signaling pathway, thus stimulating tumor growth of HNSCC. ANLN was an independent prognostic factor in patients with HNSCC. Alternatively spliced ANLN isoforms collaboratively promote HNSCC tumorigenesis in vitro and in vivo, which might provide the in-depth role and mechanism of ANLN in HNSCC development.

## Background

Head and neck squamous cell carcinoma (HNSCC) has been identified as the sixth most common cancer in the world [1, 2]. Squamous cell carcinoma (SCC) is the major pathological pattern of head and neck squamous cancer. Although improvements have been made in diagnosis and treatment strategies, patients with HNSCC at advanced stages may develop visceral metastases and present a poor prognosis with low 5-year survival rates [3–5]. It is necessary to explore the underlying molecular mechanisms of HNSCC and identify novel effective biomarkers for diagnosis and treatment of HNSCC.

Anilin actin-binding protein (ANLN) which is located on chromosome 7p14.2 and encodes an actin-binding protein containing 1125 amino acids, has been identified to play a vital role in actin cytoskeletal dynamics [6–8]. Increasing studies have shown that ANLN is highly expressed in multiple cancers, including breast cancer, ovarian cancer, kidney cancer, colorectal cancer, hepatocellular carcinoma, lung cancer, and pancreatic tumors [9–14]. Recent studies indicate that dysregulated ANLN contributes to the tumor occurrence, growth, and development. For instance, it was reported that ANLN promoted pancreatic cancer progression by regulating EZH2/miR-218-5p/LASP1 signaling axis [15]. ANLN promoted doxorubicin resistance in breast cancer cells by activating RhoA [16]. ANLN played a critical role in human lung carcinogenesis through the activation of RHOA and by involvement in the phosphoinositide 3-kinase/AKT pathway [16]. Interestingly, a study reported that the splice variants of ANLN such as ANLN-201 (ENST00000265748.6), ANLN-202 (ENST00000396068.6), ANLN-210 (ENST00000457743.1), ANLN-212 (ENST00000491782.1) were highly expressed compared to other transcripts in bladder cancer, indicating that ANLN splice variants have differential influence on tumor development [9]. Nevertheless, biological functions and regulatory mechanisms of ANLN transcripts involved in HNSCC progression are not quite clear.

Alternative RNA splicing is a critical step of posttranscriptional gene expression regulation, which could bring about various RNA isoforms from a single gene, plays pivotal roles during normal biological processes such as tissue and organ development [17, 18]. RNA splicing is a highly regulated process and accumulating evidence have shown that splicing is frequently occurred in human tumors. It is common that alternative RNA splicing owns different biological functions in cancers and might contribute to malignant cellular transformation [17, 19, 20]. Studies have suggested that RNA isoforms produced by alternative RNA splicing play key roles in tumor development and thus may provide novel potential therapeutic targets in human cancer treatment.

This study for the first time investigated the expression pattern, potential roles, and regulatory mechanisms of the two transcripts of ANLN in HNSCC, which may provide new perspectives for the treatment of HNSCC.

## Results

### ANLN transcripts are specifically expressed in HNSCC

**Fig. 1:**
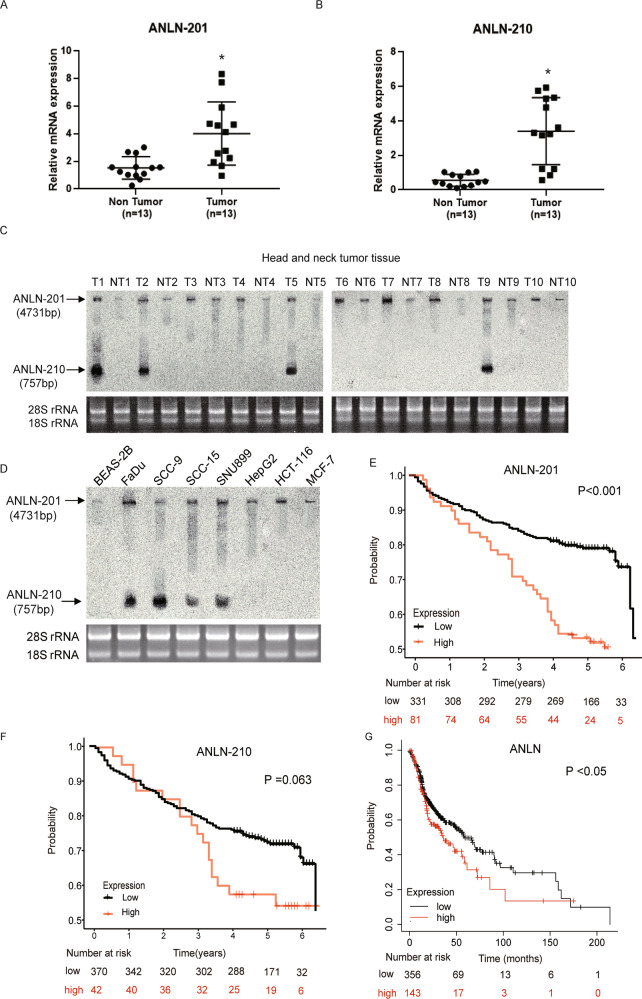
Differential expression of the two splicing isoforms of ANLN in HNSCC. **A**, **B** The mRNA levels of ANLN-201 and ANLN-210 in collected HNSCC tumor tissues and paired non-tumor tissues (*n* = 13) were analyzed by qRT-PCR. **C**, **D** ANLN-201 mRNA and ANLN-210 mRNA were detected in HNSCC tissues and several human cancer cell lines (BEAS-2B, human bronchial epithelial cells; FaDu, the hypopharyngeal carcinoma cell line; SCC-9 and SCC-15, the tongue squamous cell lines; SNU899, the laryngeal carcinoma cell line; HepG2, the hepatocellular carcinoma cell line; HCT-116, the colon cancer cell line; MCF-7, the breast cancer cell line) using Northern blot probes against ANLN-201 and ANLN-210. T: tumor tissues, NT: non-tumor tissues. **E**, **F** Kaplan–Meier plots of OS associated with ANLN-201 and ANLN-210 risk stratification in clinical trial cohort. **G** Kaplan–Meier plots of OS associated with ANLN risk stratification in external database.

To clarify the role of ANLN transcripts in HNSCC, firstly we assessed the expression levels of ANLN splice isoforms in HNSCC tumor tissues and adjacent non-tumor tissues. We observed that two splice variants of ANLN, which were ANLN-201 (ENST00000265748.6, 4731 nt) and ANLN-210 (ENST00000457743.1, 757 nt) were significantly expressed in HNSCC tumor tissues compared to the non-tumor tissues (Fig. 1A and B). Next, we examined these two ANLN transcripts in randomly selected ten pairs of HNSCC tumor tissues as shown in Fig. 1C. the expression of ANLN-201 was highly expressed in all these four pairs of tumor tissues compared to the adjacent normal tissues while ANLN-210 was expressed in tumor tissues in only two pairs of HNSCC samples. These results also indicated that ANLN-210 was not expressed in all HNSCC samples. In addition, we detected the splicing variants of ANLN in diverse tumor cell lines. Consistently, the results showed that ANLN-201 was expressed in almost all of the tested tumor cell lines whereas the ANLN-210 was only expressed in the following cell lines, including the hypopharyngeal carcinoma cell line FaDu, the two tongue squamous cell lines SCC-9 and SCC-15, and the laryngeal carcinoma cell line SNU899 (Fig. 1D). Our results suggest that ANLN-201 and ANLN-210 showed subtype-specific overexpressed in tumor cell lines and highly expressed in HNSCC cell lines such as SCC-9.

Subsequently, we analyzed the relationship between ANLN expression and clinical outcomes in 412 HNSCC samples. We found that BMI, differentiation, TNM staging and ANLN expression levels are related to overall survival (*P* = 0.011, *P* = 0.023, *P* < 0.001, and *P* < 0.001, respectively) (Table 1). Multivariate analysis showed that BMI, TNM stage, and ANLN 201 expression levels were prognostic factors for overall survival (*P* = 0.041, *P* = 0.004, and *P* = 0.037, respectively) (Table 1). Kaplan–Meier analysis showed that the upregulation of ANLN 201 expression was significantly correlated with shortened survival of HNSCC patients (*P* < 0.001, Fig. 1E). Whereas, we found that when the expression value of ANLN 210 increased, the patient’s survival period only showed a decreasing trend (*P* = 0.063, Fig. 1F). This negative association may require a larger sample size to be confirmed. In addition, according to the Kaplan–Meier chart summarized by GEO, EGA, and TCGA (http://kmplot.com/analysis/), the upregulation of ANLN was also significantly correlated with poor overall survival (*P* < 0.05, Fig. 1G).

**Table 1:** Cox proportional hazard models for prognostic factors.

	Univariate analysis		Multivariate analysis	
	HR (95% CI)	*P* value	HR (95% CI)	*P* value
Age (≥60 vs. <60)	1.34 (0.935–1.920)	0.111		
BMI (≥24 vs. <24)	0.578 (0.381–0.879)	0.011*	0.642 (0.420–0.983)	0.041*
Smoking (yes vs. no)	1.128 (0.749–1.699)	0.564		
Alcohol (yes vs. no)	1.034 (0.800–1.388)	0.796		
Differentiation (poor vs. well)	0.59 (0.374–0.931)	0.023*		
TNM stage (III + IV vs. I + II)	0.537 (0.374–0.770)	<0.001**	0.585 (0.405–0.845)	0.004**
ANLN 201 (high vs. low)	1.004 (1.002–1.005)	<0.001**	1.003 (1.000–1.006)	0.037*
ANLN 210 (high vs. low)	1.000 (1.00–1.001)	0.004**	1.000 (0.999–1.000)	0.922

*Statistically significant (*P* < 0.05), **statistically significant (*P* < 0.01).

### ANLN is critical for cell proliferation, migration, and invasion of SCC-9

**Fig. 2:**
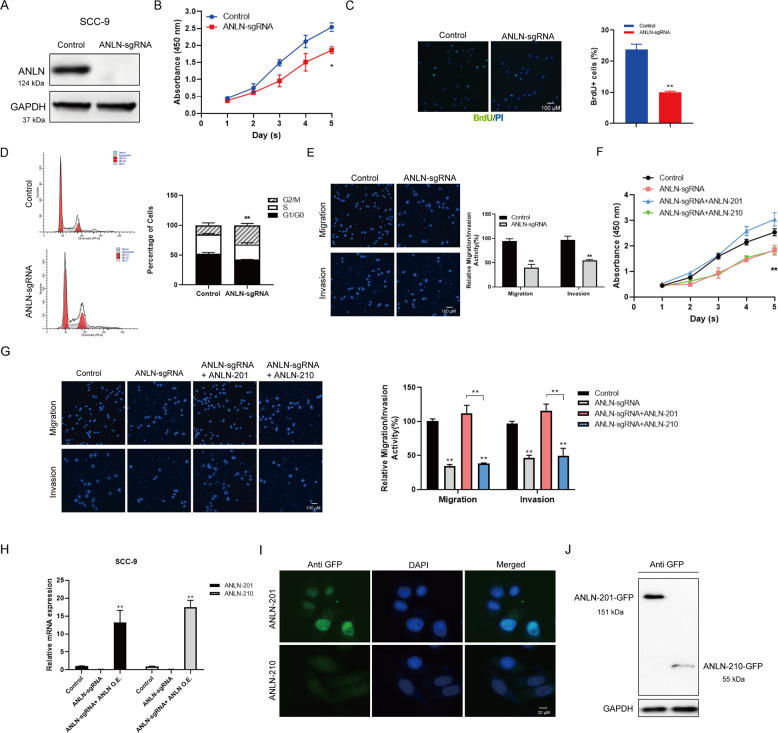
ANLN-201 promotes SCC-9 cell proliferation, migration, and invasion. **A** ANLN was knocked out in SCC-9 cells using CRISPR/Cas9. **B** Cell counting kit-8 (CCK-8) was performed to measure cell proliferation in ANLN-sgRNA SCC-9 cells. *P < 0.05. C BrdU staining together with PI was performed to measure cell proliferation in ANLN-sgRNA SCC-9 cells. *P < 0.05, **P < 0.01. **D** Cell cycle analysis was used to analyze the change of cell cycles induced by ANLN knockout. *P < 0.05, **P < 0.01. **E** Transwell and transwell-matrigel assays were performed to measure cell migration and cell invasion caused by ANLN knockout. *P < 0.05, **P < 0.01. **F** Cell proliferation was assessed in ANLN-sgRNA SCC-9 cells transfected with GFP-ANLN-201 or GFP-ANLN-210. *P < 0.05, **P < 0.01. **G** Cell migration and cell invasion were analyzed in ANLN-sgRNA SCC-9 cells transfected with GFP-ANLN-201 or GFP-ANLN-210. *P < 0.05, **P < 0.01. **H** qRT-PCR was used to examine the mRNA level of ANLN-201 or ANLN-210 in ANLN-sgRNA SCC-9 cells transfected with ANLN overexpressing plasmids GFP-ANLN-201 or GFP-ANLN-210. *P < 0.05, **P < 0.01. **I** The immunofluorescence of ANLN-201 and ANLN-210 was assessed in ANLN-sgRNA SCC-9 cells transfected with ANLN overexpressing plasmids GFP-ANLN-201 or GFP-ANLN-210 using primary antibody against GFP. **J** The protein levels of ANLN-201 and ANLN-201 were estimated by immunoblot using the antibody against GFP.

To investigate the role of ANLN in HNSCC, we established the stable ANLN-knockout cell line in SCC-9 cells by using CRISPR-Cas9 (ANLN-sgRNA SCC-9) for further study (Fig. 2A). We then assessed the effects of ANLN knockout on the cell malignancy of SCC-9. As shown in Fig. 2B–E, knockout of ANLN directly caused SCC-9 cell proliferation inhibition (Fig. 2B and C), triggered G2/M cell cycle arrest (Fig. 2D), and suppressed cell migration and invasion capabilities (Fig. 2E). To verify the biological function of these two ANLN isoforms in HNSCC, we conducted the in vitro functional assays by overexpressing ANLN-201 or ANLN-210 tagged with GFP in ANLN-sgRNA SCC-9 cells. The results showed that ANLN-201 overexpression could rescue the suppressed cell proliferation, cell migration, and cell invasion of SCC-9 cells induced by ANLN knockout (Fig. 2F, G). Nevertheless, overexpression of ANLN-210 does not have these similar effects. Next, we observed that both ANLN-201 and ANLN-210 were highly expressed at mRNA level in ANLN-sgRNA SCC-9 cells transfected with GFP-ANLN-201 or GFP-ANLN-210 (Fig. 2H). Interestingly, ANLN-210 expression at protein level was not detected or much lower expressed compared to ANLN-201 (Fig. 2I and J), suggesting that ANLN-210 mRNA may not be translated into protein effectively. These findings suggest that ANLN plays a key role in cell proliferation, migration, and invasion of SCC-9 and ANLN-201 and ANLN-210 may function at different patterns.

### ANLN-201 is critical for the protein stability of c-Myc

**Fig. 3:**
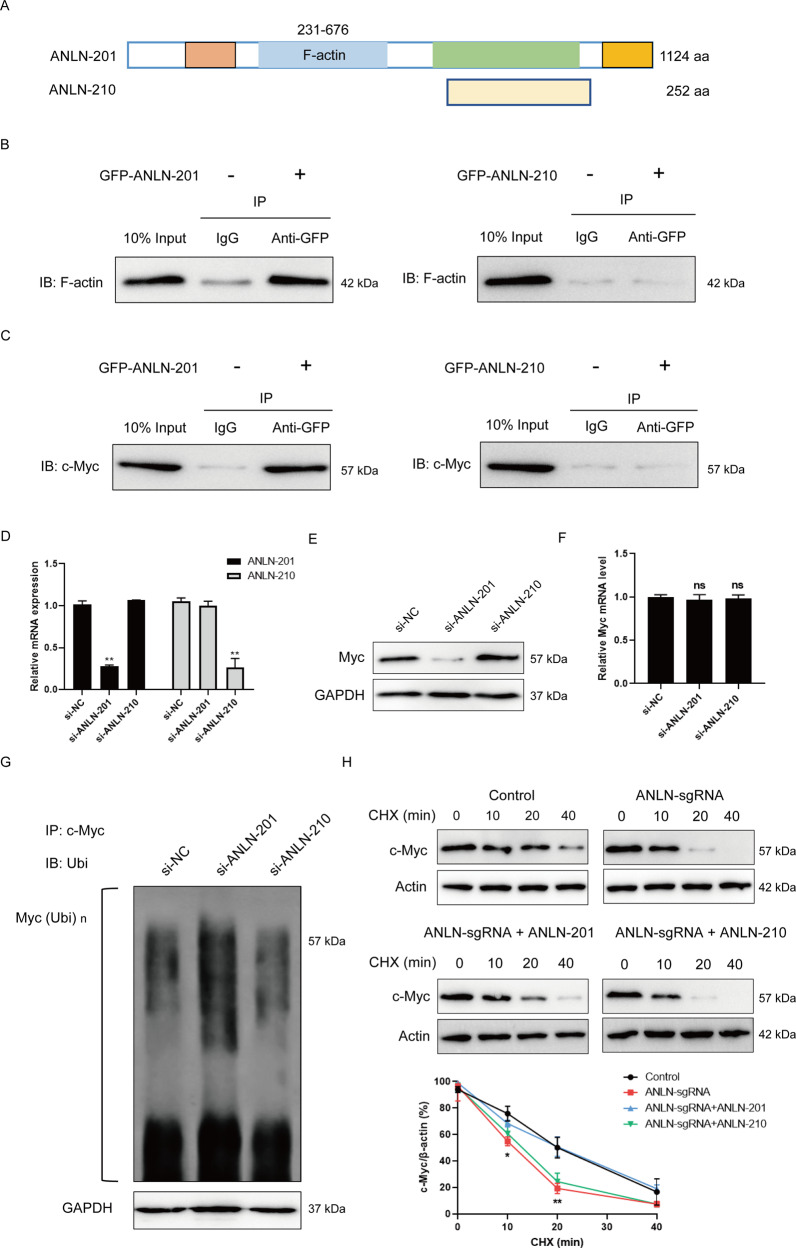
ANLN-201 interacts with Myc. **A** The sequence diagram of ANLN-201 and ANLN-210 was displayed. **B** SCC-9 cells were transfected with GFP-ANLN-201 or GFP-ANLN-210. Co-immunoprecipitation was performed between GFP-ANLN-201 or GFP-ANLN-210 and endogenous F-actin in SCC-9 cells using the <0.01. **C** Co-immunoprecipitation was performed between GFP-ANLN-210 or GFP-ANLN-201 and endogenous Myc in SCC-9 cells. **D** Transwell and transwell-matrigel assays were performed to measure cell migration and cell invasion caused by ANLN knockout. *P < 0.05, **P < 0.01. **E** Cell proliferation was assessed ed with si-ANLN-201 or si-ANLN-210. **E** The protein level of Myc was examined in si-ANLN-201 or si-ANLN-210 transfected SCC-9 cells. **F** The mRNA level of Myc was examined in si-ANLN-201 or si-ANLN-210 transfected SCC-9 cells. **G** Co-immunoprecipitation was performed in si-ANLN-201, si-ANLN-210, or si-NC transfected SCC-9 cells using antibody against Myc. Immunoprecipitates were subject to immunoblotting analysis using anti-ubiquitin. **H** SCC-9 cells were treated with 100 μM chlorhexidine (CHX) for 0, 10, 20, and 40 min in SCC-9 cells or ANLN-sgRNA SCC-9 cells transfected with GFP-control vector, GFP-ANLN-201 or GFP-ANLN-210. The protein level of Myc was detected by western blot. The relative level of Myc was determined by Image J. **P < 0.05, *P < 0.01.

It is known that there is a conserved binding domain with F-actin at N-terminal of ANLN. To further clarify the variable expression pattern and functions of ANLN alternative splicing in HNSCC in vitro, we analyzed the sequences of these two ANLN splice isoforms. The results showed that compared to the structure of ANLN-201, ANLN-210 missed the domain interacting with F-actin (Fig. 3A). The co-immunoprecipitation results also validated that GFP-ANLN-201 could interact with F-actin whereas GFP-ANLN-210 could not (Fig. 3B). It was reported that ANLN could interact with myc [21]. We confirmed that there was an interaction between GFP-ANLN-201 and Myc but not GFP-ANLN-210 with Myc (Fig. 3C). In addition, we transfected SCC-9 cells with ANLN-201 siRNA or ANLN-210 siRNA. ANLN-201/ANLN-210 mRNA expression was effectively decreased in siRNA-transfected SCC-9 cells (Fig. 3D). We observed that the expression of Myc at protein level was obviously reduced in ANLN-201-knockdown cells but not changed in ANLN-210-knockdown cells (Fig. 3E). Interestingly, neither ANLN-201 nor ANLN-210 knockdown had any effect on the expression of Myc at mRNA level (Fig. 3F). Next, we found that ANLN-201 knockdown promoted the polyubiquitination of Myc, while ANLN-210 knockdown did not have this similar effect (Fig. 3G). These results suggest that ANLN-201 mRNA but not ANLN-210 mRNA could influence the protein stability of Myc. Furthermore, we tested protein stability experiments by using protein synthesis inhibitor cycloheximide (CHX). As expected, the expression of Myc at protein level in ANLN-sgRNA SCC-9 cells was rapidly downregulated compared to the control. Overexpression of ANLN-201 could retard the protein degradation of Myc with the prolonged treating time compared to the ANLN-sgRNA cells while ANLN-210 could not (Fig. 3H). Taken together, these data indicate that ANLN-201 could interact with Myc to prevent it from degrading, and thus has an oncogenic function in SCC-9.

### HNRNPC is identified to be the RNA-binding protein of ANLN-210 mRNA

**Fig. 4:**
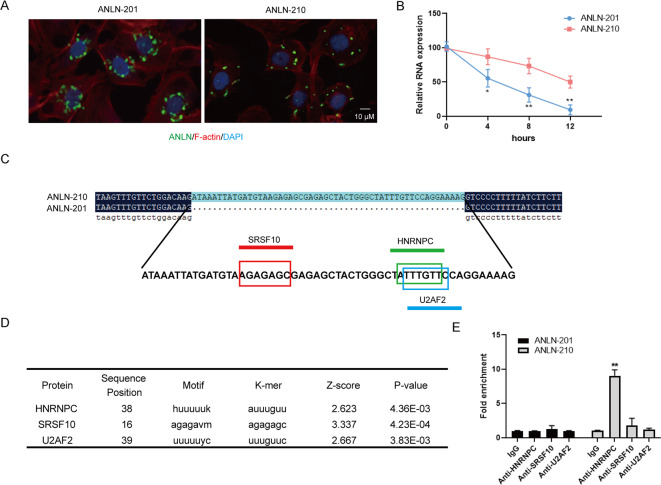
The subcellular distribution of the two splice isoforms of ANLN. **A** Immunofluorescence staining for ANLN-201 and ANLN-210 in SCC-9 cells. **B** The mRNA levels of ANLN-201 and ANLN-210 were examined in SCC-9 cells treated with 10 nM actinomycin D for 4, 8, and 12 h. **P < 0.05, *P < 0.01. **C** Sequence alignment of nucleotides between ANLN-210 and ANLN-201. **D** The predicted RNA-binding protein with ANLN-210 was listed in the table. **E** RNA co-immunoprecipitation was performed between ANLN-201/ANLN-210 and HNRNPC/SRSF10/U2AF2. *P < 0.05, **P < 0.01.

To understand the significance of the high levels of the ANLN-210 mRNA but not ANLN-210 protein, we detected the subcellular localization of the two ANLN splice variants. We found that ANLN-201 mRNA was dominantly located around the nucleus while ANLN-210 mRNA was almost distributed in the cytoplasm in ANLN-sgRNA SCC-9 cells transfected with GFP-ANLN-201 or GFP-ANLN-210 (Fig. 4A). What’s more, the RNA stability of ANLN-210 mRNA was remarkably higher than that of ANLN-201 mRNA with actinomycin D treatment (Fig. 4B). Sequence comparison analysis showed that there was extra 54 nucleotides in the sequence that encoded ANLN-210 protein compared with ANLN-201 (Fig. 4C). This extra sequence contains three potential RNA-binding protein sites, including SRSF10, U2AF2, and HNRNPC, analyzed by RBPmap online tool (http://rbpmap.technion.ac.il/) (Fig. 4D). Next, we validated that the predicted protein hnRNPC could effectively bind to ANLN-210 mRNA by RIP assay (Fig. 4E). These data reveal that ANLN splicing isoforms have different subcellular localization and distinct existing patterns.

**Fig. 5:**
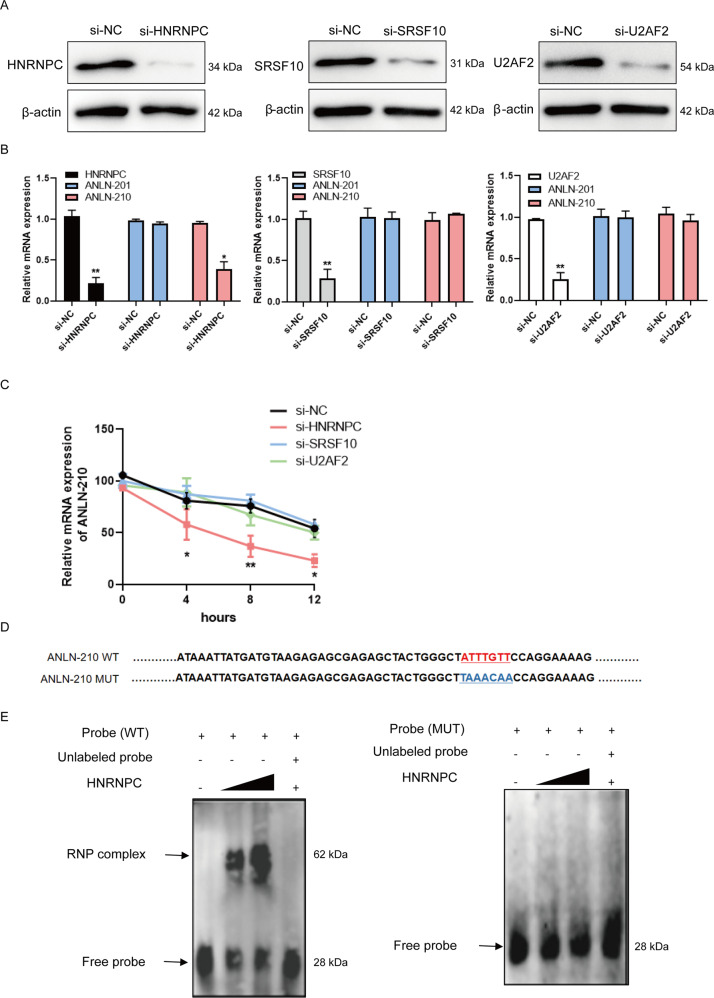
ANLN-210 interacts with HNRNPC. **A** The knockdown efficiency of si-HNRNPC, si-SRSF10, and si-U2AF2 was assessed at protein level by western blot. **B** The RNA levels of ANLN-201 or ANLN-210 were measured in si-HNRNPC, si-SRSF10, and si-U2AF2 transfected cells by qRT-PCR. *P < 0.05, **P < 0.01. **C** The RNA stability of ANLN-210 was evaluated in si-HNRNPC or si-SRSF10 or si-U2AF2 treated with actinomycin D after 4, 8, and 12 h. *P < 0.05, **P < 0.01. **D** Schematic representation of the ANLN-210 WT and ANLN-210 MUT sequences used as probes in EMSA. MUT: The binding sequences between ANLN-210 and HNRNPC were mutant. ANLN-210 MUT could not interact with HNRNPC. **E** SCC-9 cells were transfected with HNRNPC in a dose-dependent manner. EMSA was performed using ANLN-210 WT probe (left) and ANLN-210 MUT probe (right).

To further explore the relationship between ANLN-210 mRNA and HNRNPC, we performed the experiments by using si-HNRNPC, si-SRSF10, and si-U2AF2. The expression of these three predicted RNA-binding proteins HNRNPC, SRSF10 and U2AF2 was obviously decreased at mRNA and protein level (Fig. 5A and B). As expected, the relative level of ANLN-210 mRNA was reduced only in the HNRNPC-knockdown cells, while neither SRSF10 nor U2AF2 knockdown had such effects (Fig. 5B). In addition, decreased HNRNPC expression accelerated instability of ANLN-210 mRNA in SCC-9 cells with actinomycin D treatment compared to the other three groups (Fig. 5C). We subsequently constructed the ANLN-210 mutant probe (ANLN MUT), of which the binding sites to HNRNPC were mutated (Fig. 5D). As shown in Fig. 5E, the expression of ribonucleoprotein (RNP) complex could be detected in cells co-treated with ANLN WT probe and HNRNPC and enhanced with the increased transfection concentration of HNRNPC, whereas the RNP complex disappeared with ANLN-210 MUT group (Fig. 5E). Above all, our results demonstrate that HNRNPC is the RNA-binding protein of ANLN-210 mRNA and thus ANLN-210 mRNA dominantly exists in the cytoplasm of SCC-9 steadily.

Interestingly, ANLN-210 expression was upregulated at protein level in ANLN-sgRNA and HNRNPC-knockdown SCC-9 cells transfected with GFP-ANLN-210 (Figure S1A and S1B). These findings suggest that HNRNPC may keep ANLN-210 mRNA stability by hindering its translation. Consistently, HNRNPC was highly expressed in HNSCC tissues and cell lines (Figure S1C-S1E). Besides, neither ANLN-201 nor ANLN-210 knockdown in SCC-9 cells could influence HNRNPC expression at mRNA and protein levels (Figure S1F and S1G). However, ANLN-210 knockdown promoted HNRNPC localized more in the nucleus (Figure S1H). The immunofluorescence results further confirmed that HNRNPC was located in the nucleus and cytoplasm in control group SCC-9 cells; while almost distributed in the nucleus in ANLN-sgRNA cells (Figure S1I). ANLN-210 overexpression could reverse the ANLN-knockout induced the changed subcellular distribution of HNRNPC but not ANLN-201 (Figure S1I). The above results indicate that HNRNPC protects ANLN-210 mRNA stability, and in turn, ANLN-210 affects the cellular localization of HNRNPC.

### Exosomal ANLN-210 mRNA promotes M2 polarization of macrophages

**Fig. 6:**
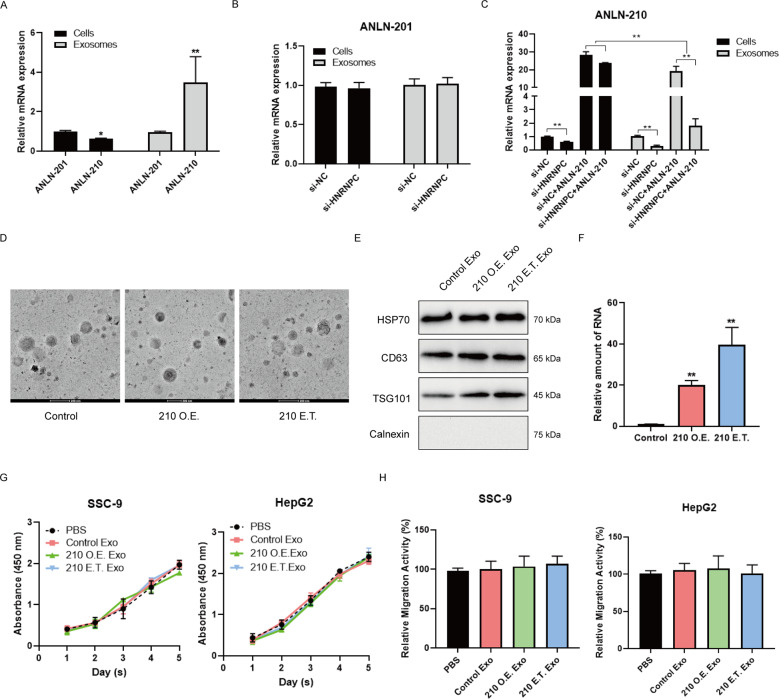
Expression pattern and functional effects of two ANLN splicing isoforms in exosomes. **A** The relative expression of ANLN-201 and ANLN-210 was examined in cells and exosomes by qRT-PCR. *P < 0.05, **P < 0.01. **B** The expression of ANLN-201 at mRNA level was examined in SCC-9 cells and exosomes when SCC-9 cells were transfected with si-HNRNPC. **C** The expression of ANLN-210 at mRNA level was examined in SCC-9 cells and exosomes when cells were transfected with si-HNRNPC and/or ANLN-210 overexpression. *P < 0.05, **P < 0.01. **D** The representative images of exosomes. Exosomes were extracted from SCC-9 cells transfected with ANLN-210 (210 O.E.) or exosomes were electrotransfected with ANLN-210 (210 E.T.). **E** Exosomal markers HSP70, CD63, and TSG101 were immunblotted in isolated exosomes. Calnexin which was expressing in cell lysates was used as the control. **F** The relative expression of ANLN-210 was examined in control, 210 O.E Exo. and 210 E.T. Exo groups. **G** Cell proliferation was performed in SCC-9 cell and HepG2 cells with the following treatment. PBS, exosomes derived from ANLN-sgRNA cells (Control Exo), exosomes derived from cells transfected with ANLN-210 (210 O.E. Exo), and exosomes electrotransfected with ANLN-210 (210 E.T. Exo). **H** Cell migration was analyzed in SCC-9 cells treated with the following treatment (PBS, Control Exo, 210 O.E. Exo, and 210 E.T. Exo).

It has been reported that the ANLN 210 mRNA was found in colon cancer cells secreted exosomes [22]. We analyzed the expression of the two ANLN transcripts ANLN-210 and ANLN-201 in SCC-9 cells secreted exosomes. There was no significant difference in expression between ANLN-201 mRNA and ANLN-210 mRNA in SCC-9 cells whereas the expression of ANLN-210 mRNA was much more higher than ANLN-201 mRNA in SCC-9 cells secreted exosomes (Fig. 6A). What’s more, the expression of ANLN-201 mRNA was not affected in HNRNPC-knockdown cells secreted exosomes compared to the control (Fig. 6B) while ANLN-210 mRNA expression in exosomes was dramatically reduced when HNRNPC expression was decreased in SCC-9 cells (Fig. 6C). Considering that HNRNPC promoted ANLN-210 mRNA stability and restained its translation, we also performed the experiments by overexpressing ANLN-210 in HNRNPC-knockdown cells. The results showed that the ANLN-210 overexpression enhanced the content of ANLN-210 mRNA in cells despite HNRNPC was knockdown whereas the expression of ANLN-210 mRNA was still downregulated in exosomes (Fig. 6C). These data indicate that HNRNPC could not only maintain the stability of ANLN-210 mRNA, but also promote the release of ANLN-210 mRNA in exosomal forms. Next, we prepared ANLN-210 mRNA enriched in exosomes in two ways. One is to transfect GFP-ANLN-210 into SSC-9 cells (210 O.E.). The other way is to electroporate GFP-ANLN-210 directly into SSC-9-derived exosomes (210 E.T.) (Fig. 6D and E). We observed that the expression of ANLN-210 mRNA was significantly increased in ANLN-210-electroporated exosomes compared to the ANLN-210-transfected group (Fig. 6F). However, these prepared exosomes rich in ANLN-210 mRNA neither accelerated SCC-9 cell proliferation and migration after co-incubation with SCC-9 cells nor promoted the hepatocarcinoma cell line HepG-2’s malignant features (Fig. 6G and H).

**Fig. 7:**
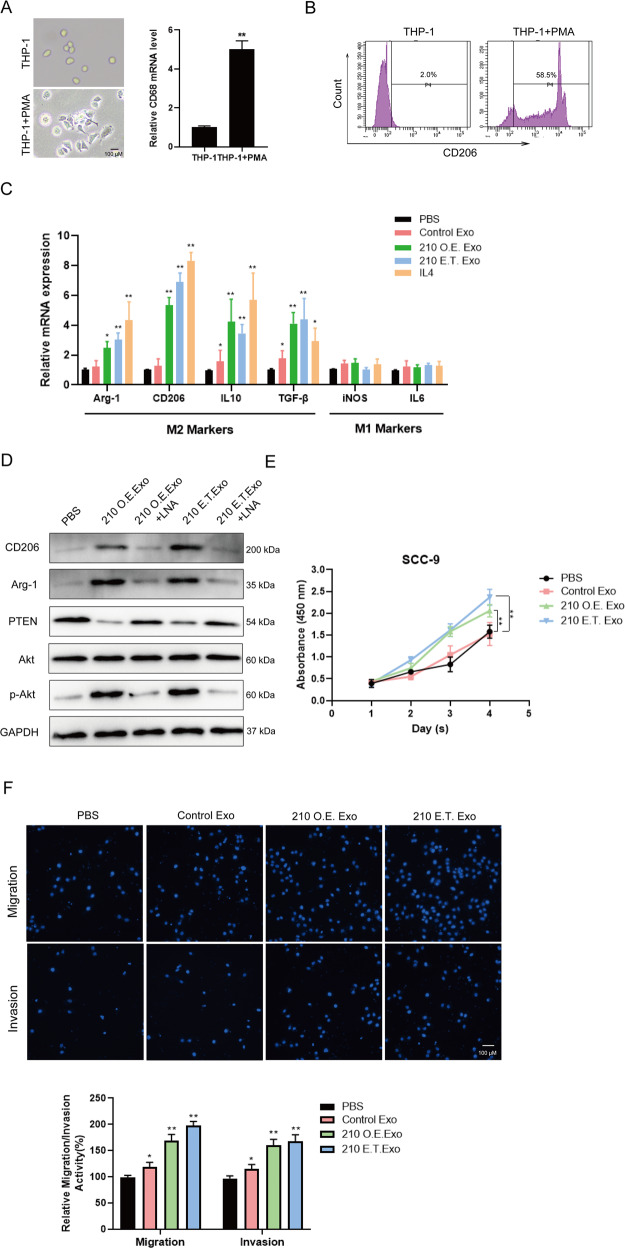
Exosomal ANLN-210 promotes M2 polarization of macrophages. **A** The representative cell morphologic photographs of macrophages. THP-1 monocytes were differentiated into macrophages using 150 nM Phorbol 12-myristate 13-acetate (PMA). The CD68 mRNA level was examined in THP-1 and PMA-induced macrophages (THP-1 + PMA). **B** Detection of macrophage marker CD206 by flow cytometry. **C** The relative expression of Arg-1, CD206, IL-10, TGF-β, iNOS, and IL-6 was measured using qRT-PCR in the following groups (PBS, Control Exo, 210 O.E. Exo, 210 E.T. Exo, and IL-4) “M2” macrophages markers: Arg-1, CD206, IL-10, and TGF-β. “M1” macrophages markers: iNOS and IL-6. *P < 0.05, **P < 0.01. **D** Western blot assays were performed to determine the protein levels of M2 markers CD206 and Arg-2, PTEN, p-Akt, Akt in macrophages treated with PBS, 210 O.E. Exo, 210 O.E. Exo + LNA, 210 E.T. Exo, 210 E.T. Exo + LNA. LNA: ANLN-210 locked nucleic acid. **E** Cell proliferation was analyzed in SCC-9 cells treated with macrophages incubated with PBS, Control Exo, 210 O.E. Exo, and 210 E.T. Exo. *P < 0.05, **P < 0.01. **F** Cell migration and invasion were analyzed in SCC-9 cells treated with macrophages incubated with PBS, Control Exo, 210 O.E. Exo, and 210 E.T. Exo. *P < 0.05, **P < 0.01.

Multiple studies have shown that tumor cells secrete exosomes to accelerate tumor growth by transferring signaling factors to the surrounding cells such as tumor-associated macrophages. To clarify the potential effects of exosomal ANLN-210 mRNA on macrophages, firstly we induced human leukemia mononuclear cell lines THP-1 to macrophages with PMA treatment. Macrophage-like morphological changes were observed in PMA-treated THP-1 cells. The expression of macrophage marker CD68 at mRNA level was significantly increased in THP-1 cells treated with PMA, indicating that THP-1 cells were successfully induced to the M0-state macrophages (Fig. 7A). In addition, we continued to use flow cytometry to determine the markers of macrophages. When THP-1 was treated with PMA, the expression of CD206 increased significantly, reaching 2.0% and 58.5%, respectively (Fig. 7B). Next, we incubated these M0-state macrophages with IL-4, PBS, or exosomes derived from SCC-9-control and SCC-9-210 O.E., or 210 E.T exosomes. Then we analyzed the expression of M2 indicators including Arg-1, CD206, IL-10, and TGF-β together with the M1 markers including iNOS and IL-6 as shown in Fig. 7C. Exosomes from SCC-9-210 OE or 210 E.T. exosomes enhanced the expressions of Arg-1, CD206, IL-10, and TGF-β while had no effect on iNOS and IL-6 expression compared with incubation with PBS or exosomes from SCC-9-control cells (Fig. 7C). Moreover, with locked nucleic acid (LNA) of anti-ANLN-210 mRNA treatment, the M2 polarization effects caused by exosomal ANLN-210 mRNA could be obviously blocked (Fig. 7D). These results suggest that exosomal ANLN-210 mRNA could promote macrophage M2 polarization.

PI3K/Akt signaling pathway is reported to be involved in regulating the polarization of macrophages. To investigate the mechanism by which exosomal ANLN-210 mRNA mediates M2 polarization, the changes of PTEN, p-Akt were detected. We observed that the PTEN expression was downregulated while p-Akt expression was upregulated in 210 O.E. exosomes and 210 E.T. exosomes treated cells. The LNA targeting ANLN-210 mRNA could significantly rescue these effects induced by exosomal ANLN-210 mRNA (Fig. 7D). Next, we examined the effects of M2 macrophages induced by ANLN-210-rich exosomes on the proliferation, migration, and invasion of SCC-9 cells. The results showed that, M2 macrophages induced by ANLN-210-rich exosomes could significantly promote SCC-9 cells proliferation compared to the control exosomes derived from the ANLN-sgRNA cells (Fig. 7E), which has the similar effects on cell migration and invasion (Fig. 7F). The above results reveal that ANLN-210 mRNA released in the form of exosomes secreted from SCC-9 could activate macrophages through PTEN/PI3K/Akt signaling pathway, leading to M2 polarization thus promotes SCC-9 cell proliferation, migration, and invasion in a feedback manner.

### Alternatively spliced ANLN isoforms synergistically promote tumor progression in vivo

**Fig. 8:**
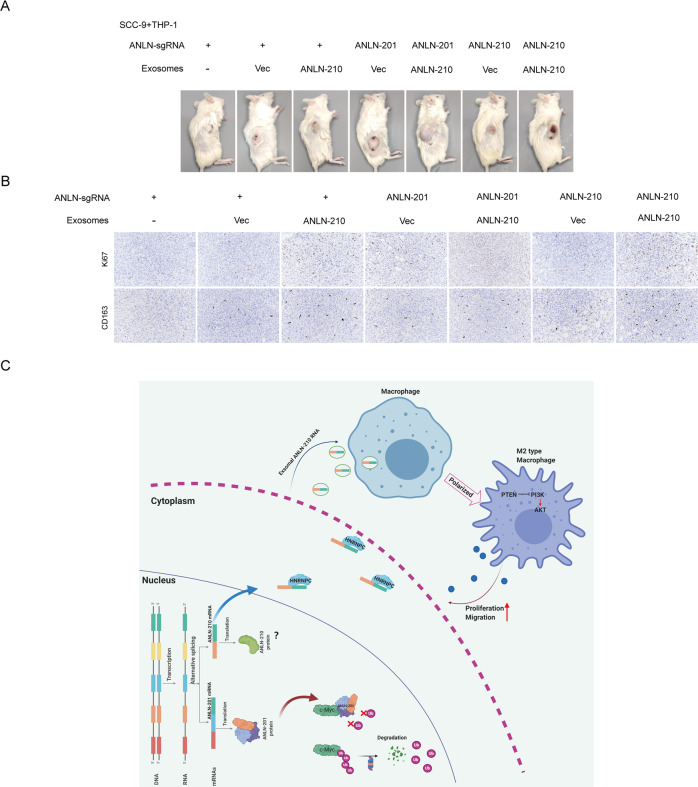
Alternatively spliced ANLN isoforms synergistically promote HNSCC tumor growth in vivo. **A** Representative images of HNSCC tumors from mice co-injected SCC-9 cells with THP-1 cells (induced by PMA) treated with exosomes (4:1) via the tail veins (*n* = 3). The groups were as follows. ① ANLN-sgRNA SCC-9 cells and THP-1 (induced by PMA) treated with PBS, ② ANLN-sgRNA SCC-9 cells and THP-1 (induced by PMA) treated with exosomes secreted from ANLN-sgRNA SCC-9 cells, ③ ANLN-sgRNA SCC-9 cells and THP-1 (induced by PMA) treated with ANLN-210 O.E. exosomes ④ ANLN-201-overexpressing SCC-9 cells and THP-1 (induced by PMA) cells treated with exosomes secreted from ANLN-sgRNA SCC-9 cells, ⑤ ANLN-201-overexpressing SCC-9 cells and THP-1 (induced by PMA) cells treated with ANLN-210 O.E exosomes, ⑥ ANLN-210-overexpressing SCC-9 cells and THP-1 (induced by PMA) cells treated with exosomes secreted from ANLN-sgRNA SCC-9 cells, and ⑦ ANLN-210-overexpressing SCC-9 cells and THP-1 cells (induced by PMA) treated with ANLN-210 O.E exosomes. **B** Representative immunohistochemistry analysis of the expression of CD163 and Ki67 in the xenograft tumor tissues. The groups were listed as in (**A**). **C** Schematic illustration of the regulatory mechanism of two ANLN splicing isoforms in the promotion of HNSCC progression.

To further evaluate the role of the two splicing isoforms of ANLN in tumor development, we co-injected ANLN-sgRNA SCC-9 cells transfected with GFP-ANLN-201 or GFP-ANLN-210 or control, and macrophages treated with ANLN-210 mRNA-rich exosomes into the nude mice. As shown in Fig. 8A, the tumor volumn of mice xenografts was increased significantly. Among them, the tumor volume in the group that ANLN-sgRNA SCC-9 cells overexpressing ANLN-201 mixed with macrophages treated with ANLN-210 mRNA-rich exosomes was the most significantly increased (Fig. 8A). Consistently, Ki67-positive together with CD163-positive stainings were the most in the group that ANLN-sgRNA SCC-9 cells overexpressing ANLN-201 mixed with macrophages treated with ANLN-210 mRNA-rich exosomes (Fig. 8B). Taken together, the results reveal that the two splicing isoforms of ANLN synergistically promote tumor progression in vivo in different ways.

## Discussion

ANLN is expressed ubiquitously in human tissues and overexpressed in multiple human tumors. However, the expression pattern and molecular mechanism of ANLN in HNSCC remain unclear. In First of all, we proved that patients with high ANLN expression have a poor prognosis. Further research shows that ANLN at mRNA level was highly expressed in HNSCC tissues and SCC-9 cells with two splicing variants (ANLN-201 and ANLN-210). Next, we discovered that loss of ANLN inhibited cell proliferation, migration, and invasion of SCC-9 cells. Furthermore, we revealed an in-depth mechanism of these two ANLN transcripts cooperatively regulating tumorigenesis of HNSCC in two ways. That is, on one hand, Myc was identified to directly interact with ANLN-201 and keep its protein stability, thus affected cell proliferation, migration, and invasion of SCC-9 cells. On the other hand, HNRNPC was found to be an RNA-binding protein of ANLN-210 and keep RNA stability of ANLN-210. Highly expressed ANLN-210 mRNA delivered to tumor-associated macrophages (TAMs) via SCC-9 cells secreted exosomes, causing activation of macrophages polarized to M2 phenotype via the canonical PI3K/Akt signaling pathway, thus promoting tumor growth and metastasis of HNSCC. These cooperative regulations of two ANLN splicing isoforms highlighted the biological functions and regulatory mechanism of ANLN in HNSCC tumorigenesis, which might provide new perspectives for cancer therapy.

HNRNPC is one of the family members of ubiquitously expressed heterogeneous nuclear ribonucleoproteins (hnRNPs), which located in the nucleus and plays critical roles in posttranscriptional regulation including roles in alternative splicing, stability, and translation [23–25]. It was previously reported that hnRNPC regulated cancer-specific alternative cleavage and polyadenylation (APA) profiles in colon cancer, which has a critical role in cancer progression [26]. In this study, HNRNPC was predicted to be one of the RNA-binding proteins of ANLN-210. Next, the interaction between ANLN-210 and HNRNPC was confirmed by RNA-IP. What’s more, we found that HNRNPC could maintain ANLN-210 RNA stability as well as prevent its protein translation. In turn, ANLN-210 knockdown promoted HNRNPC accumulation in the nucleus. However, whether HNRNPC is a critical novel regulator of cancer-specific APA for ANLN-210 in HNSCC remains to be explored.

It is known that tumor growth and progression are strongly associated with tumor microenvironment, of which solid tumors infiltrate around immune cells. Macrophages, called tumor-associated macrophages (TAMs) are rich in tumor tissues. Multiple studies have shown that macrophages can affect various aspects of tumor development. M2 macrophages play a pivotal role in promoting tumor growth, metastasis, and angiogenesis [27, 28]. Previous studies reported that tumor-associated macrophages of the M2 phenotype contributed to progression in gastric cancer with peritoneal dissemination [29]. In bladder cancer, tumor-infiltrating M2 macrophages driven by specific genomic alterations were associated with prognosis [30]. In ovarian cancer cells, miR-21 modulated the polarization of macrophages and increased the effects of M2 macrophages on promoting the chemoresistance of ovarian cancer [31]. In this study, we observed that ANLN-210 overexpression by exosomes activated macrophage to the M2 phenotype as marked by the increased expression of M2 markers Arg-1, CD206, IL-10, and TGF-β. PI3K/Akt signaling pathway has been widely recognized in multiple cell types and functions and emerged as canonical and central regulators of macrophages activation [32]. In this study, we found that exosomal ANLN-210 overexpression regulated M2 polarization by inhibiting the common target PTEN via activating the PI3K/Akt signals.

Tumor-derived exosomes, which are found in all body fluids delivering molecular and genetic messages from tumor cells to normal or other abnormal cells around. Tumor-derived exosomes play key roles in the oncogenic transformation, which have biological and clinical significance for cancer development, cancer progression, and even cancer therapy [33]. The attributes of tumor-derived exosomes might be packaged proteins, lipids, or nucleic acids, which could be regarded as biomarkers for cancer diagnosis, prognosis, and monitoring treatment responses [34, 35]. In 2008, a study reported that there were 4700 distinct mRNAs selectively packaged in exosomes derived from glioblastoma samples [36, 37]. It is well known that mRNAs carried by exosomes are involved in critical cellular activities such as cell proliferation, migration, invasion, metastasis, EMT, and so on. The present study demonstrates that exosomal ANLN-210 mRNA secreted by SCC-9 promotes macrophages polarization by targeting PTEN via PI3K/Akt. Furthermore, M2 macrophages promoted tumor growth and metastasis of HNSCC. However, it remains to be investigated whether ANLN-210 induces the activation of the PI3K/Akt pathway in the form of RNA or protein.

In summary, our findings demonstrate that the coordinate regulatory networks of alternatively spliced ANLN isoforms in HNSCC tumor growth and development, which might highlight the perspective for therapeutic strategies of HNSCC. ANLN was an independent prognostic factor in patients with HNSCC. ANLN-201 dominantly binds to Myc and exists in the nucleus in the form of protein, which can be effectively prevented from degradation and thus play a role in promoting HNSCC cell proliferation, migration, and invasion. ANLN-210 is found to bind mainly to HNRNPC, maintains its RNA stability, and secreting into the environment in the form of exosomes. Exosomal ANLN-210 mRNA promotes macrophage polarization via PTEN/PI3K/Akt signaling pathway, which in turn facilitates HNSCC tumor growth (Fig. 8C). Thus, the splicing variants of ANLN collaboratively regulate HNSCC tumorigenesis in two ways.

## Materials and methods

### Patients and cancer tissues

This study was approved by the Ethics Committee of Harbin Medical University, China. All patients provided written informed consent to participate in the study. The research methodology meets the standards set out in the Declaration of Helsinki. From January 2013 to December 2015, 412 HNSCC tissue samples and some normal tissues were collected from the Affiliated Cancer Hospital of Harbin Medical University. All tumor tissues were independently diagnosed as HNSCC by two pathologists. Patients receiving chemotherapy or radiation therapy were excluded. Overall survival (OS) is defined as the time interval between the date of definitive diagnosis and the date of death or the last follow-up.

### Cell lines and cell culture

Human HNSCC lines SCC-9, SCC-15, and FaDu were grown in DMEM/F12 (Invitrogen, Carlsbad, CA, USA) containing 10% fetal bovine serum (FBS, Gibco). Human bronchial epithelial cell line BEAS-2B was cultured in DMEM supplemented with 10% LHC-9 medium (Invitrogen) and 10% FBS (Gibco). Human hepatocellular carcinoma cell line HepG2, human colorectal cancer cell line HCT-116, and breast cancer cell line MCF-7 were cultured in DMEM supplemented with 10% FBS. Laryngeal squamous cell carcinoma cell line SNU899 was maintained in RPMI-1640 (Gibco) with 10% FBS (Gibco). Human monocytic THP-1 cells were maintained in RPMI-1640 containing 10% of heat-inactivated FBS and supplemented with 10 mM Hepes (Gibco), 1 mM pyruvate (Gibco), 2.5 g/l D-glucose (Merck), and 50 pM ß-mercaptoethanol (Gibco). THP-1 monocytes were differentiated into macrophages incubated with 150 nM PMA (Sigma) followed for 24 h. These cancerous cell lines were maintained in a humidified incubator at 37 °C with 5% CO_2_.

### Quantitative real-time PCR (qRT-PCR)

Total RNA was extracted with TRIzol reagent and reverse transcribed to cDNA using a PrimeScript RT reagent kit (TaKaRa, Tokyo, Japan) according to the manufacturer’s instructions. Quantitative real-time PCR (qRT-PCR) was performed using the SYBR Green qPCR Master Mix (Takara). The sequences of primers were listed in Table 2.

**Table 2:** Primers of gene for qRT-PCR.

Genes	Sequences
GAPDH	Forward: 5′-TGGATTTGGACGCATTGGTC-3′
	Reverse: 5′-TTTGCACTGGTACGTGTTGAT-3′
ANLN-201	Forward: 5′-ATGTCTTCGTGGCCGATTTGA-3′
	Reverse: 5′-CTCTGACAGTGAGTTTCCTGTTT-3′
ANLN-210	Forward: 5′-TGCCAGGCGAGAGAATCTTC-3′
	Reverse: 5′-CGCTTAGCATGAGTCATAGACCT-3′
MYC	Forward: 5′-CTTTCCTCCACTCTCCCTGG-3′
	Reverse: 5′-AACCCTCTCCCTTTCTCTGC-3′
HNRNPC	Forward: 5′-GGAGATGTACGGGTCAGTAACA-3′
	Reverse: 5′-CCCGAGCAATAGGAGGAGGA-3′
SRSF10	Forward: 5′-TGAGGATGTTCGTGATGCTGA-3′
	Reverse: 5′-CCTCCTTTCATAACTTCGGCTT-3′
U2AF2	Forward: 5′-ACCCAGGCTATGGCCTTTG-3′
	Reverse: 5′-GAAGCGGCTGGTAGTCGTG-3′

### Northern blot

RNA was extracted and heat denatured at 65 °C for 15 min. The prepared RNA samples were loaded in the gel in the MOPS running buffer, which was electrophoresed at 5–6 V/cm until the bromophenol blue (the faster-migrating dye) migrated at least 2–3 cm into the gel. The gel was visualized on a UV transilluminator. The 28S rRNA band should be approximately twice as intense as the 18S rRNA band.

### Cell cycle analysis

The harvested SCC-9 cells were re-suspended with PBS and fixed overnight with 70% ethanol at 4 °C, then incubated with PI staining reagent at room temperature for 30 min. The treated cells were analyzed using a FACS Calibur system (BD Biosciences, San Jose, CA, USA).

### Western blot

Proteins were extracted from tissues and cells. The protein concentration was determined by the bicinchoninic acid (BCA) Kit (Beyotime, Shanghai, China). Proteins were separated with 10% sodium dodecyl sulfate-polyacrylamide gel electrophoresis (SDS-PAGE) and transferred to the nitrocellulose membrane. After blocking, the membranes were incubated in primary antibodies at 4 °C overnight. ANLN-201 and ANLN-210 (1:1000, GeneX Health, Beijing), ANLN (1:1000, ab211872, Abcam, Cambridge, MA, USA). Myc (ab32072), F-actin (ab130935), HNRNPC (ab75822), SRSF10 (ab254935), U2AF2 (PA5-30442, Invitrogen), HSP70 (ab2787), CD63 (ab134045), TSG101 (ab125011), Calnexin (ab22595), CD206 (ab125028), Arg-1 (ab133543), PTEN (ab267787), Akt (ab8805), p-Akt (ab38449), GAPDH (ab9485), and β-actin (ab8226), GAPDH and β-actin was used as the internal control. Protein bands were visualized by chemiluminescence (ECL, Forevergen, Guangzhou, China). The experiment was conducted for three times.

### Immunoprecipitation

To detect ANLN associated with F-actin/Myc, total protein was extracted from SCC-9 cells after treatment. Cell lysates for immunoprecipitation were pre-cleared and incubated with GFP-protein-magnetic beads overnight at 4 °C. The beads were washed and boiled with loading buffer, then for immunoblots with GFP (Abcam) antibodies.

### Isolation of exosomes

Exosomes were isolated from cell culture medium by ultracentrifugation according to the previous reports [38]. The procedures were performed at 4 °C. Briefly, cells were removed by centrifugation at 300 × *g*. Other debris were removed by centrifugation at 3000 × *g*. After that, the supernatants were centrifuged at 10,000 × *g* for 30 min to remove shedding vesicles and others. At last, the supernatants were centrifuged at 110,000 × *g* for 70 min. Exosomes were obtained from the pellets and re-suspended in PBS.

### Cell proliferation analysis

Cell proliferation of SCC-9 cells was determined using the Cell Counting Kit-8 (CCK-8; Dojindo Laboratories, Kumamoto, Japan) according to the manufacturer’s instructions. SCC-9 cells transfected as experiments designed were plated in 96-well plate. Cell viability was analyzed for 24, 48, 72, and 96 h. CCK-8 was added and incubated for 3 h according to the time points. The absorbance was measured at 450 nm.

### Cell migration and invasion assays

Cell migration and invasion assays were performed using transwell insert chambers (BD Biosciences, USA). SCC-9 cells were transfected as experiments designed. The transfected SCC-9 cells in serum-free medium were seeded into the top chamber. The medium containing 20% FBS was added to the lower chambers. After routinely cultured for the appropriate time, cells on the lower membrane surface of the top chamber were fixed with methanol and stained with DAPI. For invasion analysis, the top chambers were coated with the matrigel (BD Biosciences, USA).

### Immunofluorescence

SCC-9 cells were fixed and permeabilized, then incubated with GFP Rabbit antibody (ab290, Abcam) for 3 h at 4 °C. After washing three times with PBS, the cells were incubated with Alexa Fluor 488 conjugated Goat Anti-Rabbit IgG (H&L) (ab150077; Abcam) for 1 h at room temperature in the dark place. After washing three times with PBS, cells were treated with DAPI (D9564, Sigma) for 10 min at room temperature. Cells were photographed using the confocal microscope (Olympus FV-1000).

### Gel electrophoretic mobility shift binding assay (EMSA)

Protein-RNA interactions were evaluated using the LightShift Chemiluminescent RNA EMSA kit (Thermo Scientific). The constructed T7 promoter-ANLN-210 WT or ANLN-210 MUT DNA fragments were used as the templates for in vitro transcription to synthesize plenty of RNA. The 3′end of synthesized RNA was biotinylated using RNA 3′End Biotinylation Kit (Pierce, Rockford, IL, USA), which was used as the probe analyzed for EMSA. The recombinant HNRNPC protein was obtained from Origene (TP315956). Biotin-labeled RNA and a dose-dependent amount of HNRNPC were mixed in the reaction buffer. The samples were electrophoresised on the 5% nondenaturing polyacrylamide gel. The signals were detected using the Luminescent image analyzer (FluorChem M).

### Immunohistochemistry (IHC)

Paraffin-embedded HNSCC tissues were fixed in 4% polyformaldehyde (PFA). IHC staining was performed according to the manufacturer’s protocol. The deparaffinized and rehydrated histologic sections were aimmersed in 10 mM citrate buffer for heat-induced antigen retrieval. The slides were stained using antibodies against CD163 (ab182422, Abcam) and Ki67 (ab15580, Abcam).

### Animals

Male BALB/c nude mice (6–8 weeks) were raised strictly in the pathogen-free animal house in accordance with feeding standards, which was approved by the Institutional Animal Care and Research Advisory Committee of Affiliated Tumor Hospital of Harbin Medical University.

### Xenograft models

To assess the effects of exosomal ANLN-210-activiated M2 polarized macrophages on HNSCC tumor growth in vivo, 1.5 × 10^7^ SCC-9 cells were mixed in a 4:1 ratio with the macrophages transfected with exosomes with different treatment as experiment designed. Then the cell mixture was co-injected into the flanks of the nude mice. After 3 weeks, the mice were euthanized and the tumors were obtained and fixed in formaldehyde for and IHC staining.

### Statistical analysis

All the experiments were repeated at least three times. The data were analyzed using SPSS 20.0 and GraphPad Prism 7. The *P*-value <0.05 was considered to be significant.

## Supplementary information

Figure S1

## Data Availability

The datasets generated and analyzed during the current study are available from the corresponding author on reasonable request.
