# Supplementary material for: Alternatively spliced ANLN isoforms synergistically contribute to the progression of head and neck squamous cell carcinoma
Source: Cell Death Dis. 2021 Aug 3;12(8):764. doi: 10.1038/s41419-021-04063-2 (PMC8333361; doi:10.1038/s41419-021-04063-2)
Supplement: Supplementary file 1 — Figure S1 [file 41419_2021_4063_MOESM1_ESM.docx]

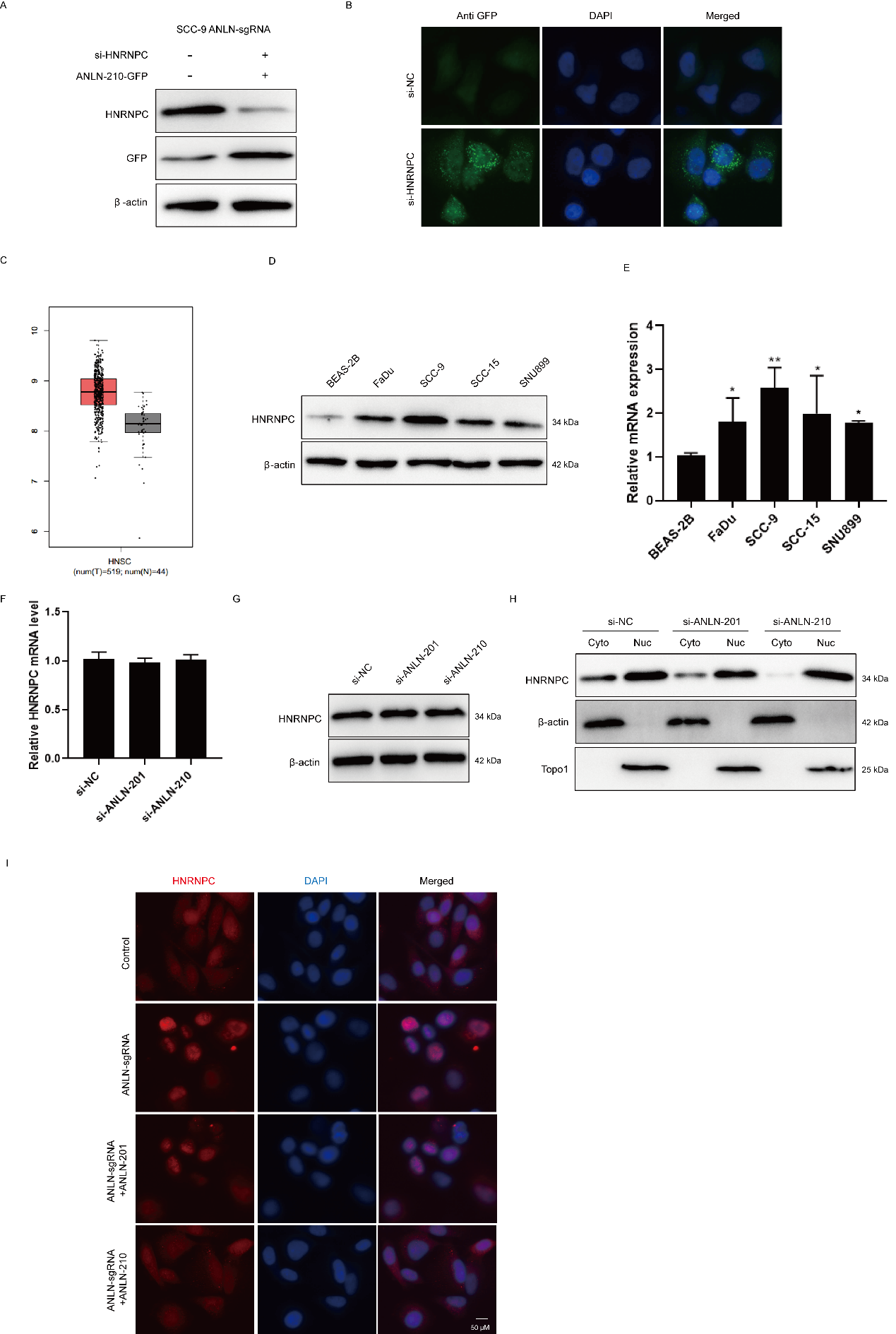


**Figure S1** Relationship between ANLN-210 and HNRNPC in HNSCC tissues and cell lines. (A and B) The protein level and subcellular distribution of ANLN-210 was examined in ANLN-sgRNA SCC-9 cells co-transfected with si-HNRNPC and GFP-ANLN-210. (C) The relative expression of HNRNPC was analyzed in HNSCC tumor tissues (T=519) and normal tissues (n=44). (D) HNRNPC expression at protein level was analyzed in different types of tumor cell lines BEAS-2B, FaDu, SCC-9, SCC-15 and SNU899. (E) The relative mRNA level of HNRNPC was analyzed in BEAS-2B, FaDu, SCC-9, SCC-15 and SNU899. *p＜0.05, **p＜0.01. (F and G) HNRNPC expression at mRNA and protein levels were measured in SCC-9 cells transfected with si-ANLN-201, si-ANLN-210 and si-NC. *p＜0.05, **p＜0.01. (H and I) The protein levels and subcellular distribution of HNRNPC in the nucleus and cytoplasm were analyzed in SCC-9 cells transfected with si-ANLN-201, si-ANLN-210 and si-NC.
